# Normative Data for The Dimensions and Volume of Pituitary Gland in The General Population Undergoing Magnetic Resonance Imaging Under a Tertiary Care Center: An Observational Study

**DOI:** 10.31729/jnma.9088

**Published:** 2025-06-30

**Authors:** Suman Paudel, Prerana Singh Rokaha, Pratik Singh Rokaha, Apeksha Bista, Nischal Subedi, Narayan Bikram Thapa

**Affiliations:** 1 Department of Radiology, Kathmandu Medical College and Teaching Hospital, Sinamangal, Kathmandu, Nepal; 2 Kathmandu Medical College and Teaching Hospital, Sinamangal, Kathmandu, Nepal; 3 Lumbini Medical College and Teaching Hospital, Tansen, Palpa, Nepal

**Keywords:** *magnetic resonance imaging*, *pituitary gland*, *radiology*

## Abstract

**Introduction:**

The pituitary gland is a pivotal neuroendocrine organ responsible for regulating essential physiological functions. Normative data on its dimensions and volume are crucial for identifying pathological changes in clinical settings. However, such reference values are often limited or population-specific. This study aimed to establish normative data for pituitary gland dimensions and volume in the general population undergoing magnetic resonance imaging (MRI) at a tertiary care center.

**Methods:**

A total of 870 subjects of all age groups presumed normal pituitary morphology were evaluated using magnetic resonance imaging. Mid sagittal T1 weighted image and coronal T2 weighted images on magnetic resonance imaging were used to measure height, length and width of the pituitary gland. Data was stratified into 10 groups on the basis of age group in each sex to observe the differences.

**Results:**

The height and volume of the pituitary gland showed variations with age. Positive correlation was observed between the mean height and volume for the sexes combined which was statistically significant with Pearson correlation coefficient r=0.82 (p<0.001).

**Conclusions:**

This study provides normative data for measurements of the pituitary gland and its variations based on age and sex.

## INTRODUCTION

The pituitary gland (PG), a small yet vital endocrine organ, regulates growth, metabolism, reproduction, and homeostasis.^1,2^. The PG development varies with age and sex due to fluctuating neuroendocrine changes throughout life.^3,4^

Normal and abnormal pituitary glands are usually distinguishable, but diagnostic challenges arise with physiological hypertrophy, subtle microadenomas, lobulated margins, and inflammation.^5^ Studies report mixed findings on pituitary dimensions, with some identifying age-related correlations and others observing no differences in certain groups.^3,6.7^ Magnetic Resonance Imaging (MRI), using atomic nuclei, safely provides high-resolution images for detailed soft tissue and pituitary assessment.^6,8^ These studies provide population-specific pituitary reference values, highlighting the need for age- and region-tailored normative data.

The aim of this study is to establish normative data for the dimensions and volume of pituitary gland in the general population undergoing MRI.

## METHODS

This study was a descriptive cross-sectional study conducted among the patients of all age groups undergoing MRI at a tertiary care center, Kathmandu Medical College Teaching Hospital, Nepal. For sample size, total population sampling method was used and patients of all the age groups undergoing MRI in between 1st September 2024 to 30th December 2024 in Kathmandu Medical College who fulfils the inclusion criteria were taken.

Patients from all the age groups who fulfils the inclusion criteria of the study were included in this study. Patients with pituitary pathologies, a history of intracranial surgery, radiation exposure, psychiatric illness, antipsychotic drug use, pregnancy, lactation, or any suspected or diagnosed hormonal imbalance, epilepsy or hydrocephalus were excluded from the study. Ethical clearance was obtained from the Institutional Review Board (Reference number: 12082024/17) of Kathmandu Medical College. No identifiable individual information was used and patient records were removed before being included in the analysis.

MRI examinations were performed using Siemens MAGNETOM Avanto 3 Tesla scanner. Our study considered all the parameters, such as anteroposterior dimension, height, and the transverse dimension of the pituitary, as the size and shape of a normal pituitary gland can vary according to age, gender, and the hormonal environment of the patient. The different measurements of the pituitary gland were taken from the T1 weighted image in mid sagittal section and T2 weighted image in the coronal section. Width of the pituitary gland was measured as its maximum transverse extent in the T2 coronal section. Length of the pituitary gland was measured as maximum anteroposterior diameter in Tl mid sagittal section. Height of the pituitary gland was measured as its maximum cranio-caudal extent in T1 Mid sagittal section. Volume of the pituitary gland was calculated in SPSS using the ellipsoid volume formula = length × width × height × pi/6.

We collected data using a structured proforma. Data were entered using the Microsoft Excel 2016 and IBM SPSS Statistics for Windows, version 26 (IBM Corp., Armonk, N.Y., USA) was used for analysis. Point estimate at 95% Confidence Interval was calculated. Mean and Standard deviations of pituitary height were calculated in the scale of mm. Volumes of pituitary gland were calculated in the scale of mm^3^.

Correlation study was performed to see the relationship between the pituitary height and pituitary volume.

## RESULTS

The MRI from 870 subjects with presumed normal pituitary was reviewed of which 396 were male (mean age 43.81 years) and 474 were female (mean age 44.80 years). The sex distribution of the study population is presented (Table 1).

**Table 1:** Sex distribution of the study population.

Sex	n(%)
Female	474 (54.51)
Male	396 (45.49)
Total	870 (100)

The mean pituitary height among the sample population was 4.89 +/- 1.33 mm and the mean pituitary volume was 282.08 +/- 104.31 cc, (Table 2).

**Table 2:** Dimensions of the pituitary glands in different age groups.

Age group	n(%)	Pituitary length (mm) (mean+/-SD)	Pituitary width (mm) (mean+/-SD)	Pituitary height (mm) (mean+/-SD)	Pituitary volume (cc) (mean+/-SD)
0-10	27(3.11)	7.10+/-1.93	10.67+/-1.92	3.63+/-0.97	144.56+/-59.94
11-20	40(4.62)	9.60+/-1.46	12.06+/-2.20	6.38+/-1.60	389.25+/-132.14
21-30	202(23.36)	9.40+/-1.35	12.25+/-1.50	5.39+/-0.91	325.68+/-86.49
31-40	142(16.34)	9.26+/-1.44	11.91+/-1.74	5.12+/-1.38	294.67+/-97.73
41-50	130(14.92)	9.18+/-1.23	12.11+/-1.82	4.9+/-1.46	288.50+/-114.46
51-60	142(16.32)	9.55+/-1.64	11.36+/-1.61	4.69+/-1.23	264.13+/-85.93
61-70	77 (8.91)	8.92+/-1.80	11.19+/-1.60	4.37+/-0.81	228.76+/-72.73
71-80	65(7.52)	9.46+/-1.34	11.93+/-1.84	4.02+/-1.30	237.11+/-91.57
81-90	38 (4.41)	9.16+/-1.21	11.79+/-1.67	4.03+/-1.06	230.06+/-81.20
91-100	7(0.86)	9.66+/-1.07	10.22+/-0.99	4.23+/-0.58	217.41+/-104.31
Total	870(100)	9.26+/-1.52	11.81+/-1.74	4.89+/-1.33	282.08+/-104.31

The mean pituitary height was 4.87±1.48 mm in males and 4.91 ±1.41 mm in females. Similarly, the mean pituitary volume was 274.91±94.84 mm^3^ (range: 49.01-604.55 mm^3^) in males and 288.06±111.36 mm3 (range: 49.01-604.55 mm^3^) in females. Subjects were categorized into ten age groups (0-10, 11-20, ..., 91-100) and pituitary gland size were calculated for each age groups according to sex, (Table 3 and Table 4).

**Table 3:** Dimensions of the Pituitary glands in males of different age groups.

Age group 0-10	n(%)	Pituitary length (mean+/-SD)	Pituitary width (mean+/-SD)	Pituitary height (mm) (mean+/-SD)	Pituitary volume (cc) (mean+/- SD)
0-10	19(4.82)	6.70+/-1.68	11.13+/-2.03	3.69+/-1.08	140.99+/-56.37
11-20	14(3.56)	9.83+/-1.03	10.77+/-2.96	5.60+/-0.73	302.90+/-74.10
21-30	100(25.34)	9.39+/-1.43	11.91+/-1.44	5.40+/-0.91	315.41+/-76.96
31-40	55(13.93)	9.35+/-1.50	11.29+/-1.54	4.95+/-1.36	273.24+/-88.96
41-50	56(14.11)	9.20+/-1.56	11.90+/-1.94	4.96+/-1.57	291+/-126.23
51-60	78(19.72)	9.61+/-1.22	11.05+/-1.57	4.61+/-0.94	258.38+/-79.74
61-70	29(7.31)	9.15+/-1.33	11.15+/-1.87	4.51+/-0.74	236.39+/-45.05
71-80	25(6.35)	10.40+/-1.21	12.10+/-1.69	4.30+/-1.69	276.67+/-109.11
81-90	15(3.82)	9.52+/-1.32	11.58+/-1.21	4.52+/-1.03	264.14+/-79.83
91-100	5(1.33)	9.86+/-1.22	10.38+/-1.16	4.32+/-0.66	228.70+/-31.58
Total	396 (100)	9.34+/-1.48	11.5+/-1.73	4.87+/-1.23	274.91+/-94.84

**Table 4:** Dimensions of the Pituitary glands in females of different age groups.

Age group	n(%)	Pituitary length (mean+/-SD)	Pituitary width (mean+/-SD)	Pituitary height (mm) (mean+/-SD)	Pituitary volume (cc) (mean+/-SD)
0-10	8(1.71)	8.06+/-2.27	9.58+/-1.09	3.50+/-0.66	153.06+/-71.12
11-20	26(5.52)	9.47+/-1.65	12.76+/-1.23	6.80+/-1.79	435.75+/-134.01
21-30	102(21.55)	9.42+/-1.27	12.59+/-1.50	5.37+/-0.91	335.76+/-94.20
31-40	87(18.44)	9.20+/-1.41	12.31+/-1.76	5.22+/-1.39	308.22+/-101.05
41-50	74(15.62)	9.16+/-1.29	12.27+/-1.71	4.85+/-1.37	285.97+/-105.50
51-60	64(13.51)	9.49+/-2.04	11.73+/-1.61	4.78+/-1.51	271.12+/-93.08
61-70	48(10.11)	8.78+/-2.04	11.21+/-1.42	4.29+/-0.84	223.15+/-85.37
71-80	40(8.42)	8.87+/-1.06	11.83+/-1.94	3.86+/-0.97	212.39+/-69.31
81-90	23(4.93)	8.92+/-1.10	11.83+/-1.94	3.72+/-0.97	207.84+/-75.66
91-100	2(0.41)	9.15+/-0.37	9.82+/-0.27	4.00+/-0.33	189.16+/-28.52
Total	474(100)	9.19+/-1.54	12.08+/-1.71	4.91+/-1.41	288.07+/-111.36

The female groups were further divided into reproductive (15-49) and non-reproductive groups and pituitary gland size were calculated for reproductive and non-reproductive age groups, (Table 5).

**Table 5:** Dimensions of the Pituitary glands in females of different age groups.

Age group	n(%)	Pituitary length (mean+/-SD)	Pituitary width (mean+/-SD)	Pituitary height (mm) (mean+/-SD)	Pituitary volume (cc) (mean+/- SD)
0-14	17 (3.6%)	8.45+/-1.90	11.47+/-2.35	4.99+/-2.29	287.07+/-190.27
15-49	274 (57.8%)	9.29+/-1.35	12.47+/-1.56	5.28+/-1.30	321.98+/-107.06
>=50	183 (38.6%)	9.11+/-1.75	11.56+/-1.72	4.34+/-1.27	237.37+/-86.80

**Figure 1:**
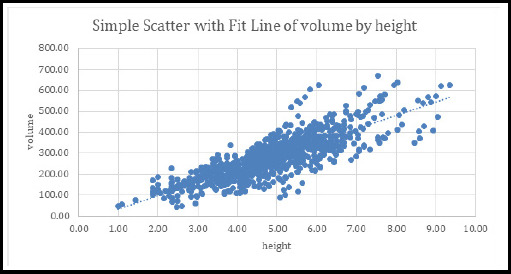
Correlation of height and volume among study population. BCVA=Best corrected visual acuity

There was positive correlation between the mean height and volume for the sexes combined which was statistically significant with Pearson correlation coefficient r=0.0.82 (p<0.001), (Figure 1).

## DISCUSSION

The pituitary gland is a small endocrine gland that sits in the sella turcica, a saddle-shaped depression in the central sphenoid bone. It is vital to the body's metabolism because it generates, stores, secretes, and regulates many kinds of key hormones in the body.^1^ The pituitary gland has two embryonic origins: the anterior and intermediate lobes emerge from the oral ectoderm, and the posterior lobe from the neural ectoderm.^2^ The pituitary gland develops sequentially, resulting in a complex organ with five cell types.^2^ The anterior pituitary gland secretes six hormones: somatotrophs (growth hormone; GH), thyrotrophs (thyrotrophin or thyroid-stimulating hormone; TSH), lactotrophs (prolactin; PRL), gonadotrophs (follicle-stimulating hormone; FSH and luteinizing hormone; LH), and corticotrophs (corticotrophin or adrenocorticotrophic hormone; ACTH).^2^ Proopiomelanocortin (POMC), a key precursor to endorphins and melanocyte-stimulating hormone (MSH), is secreted by the melanotrophs in the intermediate lobe.^2^ The posterior pituitary or neurohypophysis consists of the axonal terminals of magnocellular neurons from the supraoptic and paraventricular nuclei of the hypothalamus secreting arginine vasopressin and oxytocin, respectively.^2^

The development of the pituitary gland relies on changing neuroendocrine factors, causing its height and volume to naturally vary with age and sex throughout life.^9^ Previous research has suggested that changes in the endocrine environment can be reflected in pituitary morphology.^9^ The rise in pituitary height during puberty could be attributed to increased luteinizing hormone secretion. The higher pituitary height in young people, both male and female, may indicate physiological neuroendocrine differences between younger and older individuals. Pituitary height loss with age may also represent age-related endocrinology and physiological pituitary atrophy.^9^ However, normative data on pituitary size in individuals has shown inconsistent findings in different studies.^9-11^ Establishing clear standards is crucial for guiding clinical decisions in managing pituitary disorders.

Magnetic resonance (MR) imaging has proven to be an effective and accurate method due to its numerous advantages over other modalities.^9^ Its superior spatial and contrast resolutions enable the detection of anomalies, while its rapid temporal resolution enables the indirect assessment of its blood supply via dynamic imaging.^4^

The present study aimed to establish normative data for pituitary gland dimensions and volume in a general population undergoing MRI. Most patients whose MRI scans were analyzed belonged to the 21-30-year age group, differing from the findings of Shajil et al. and Khanal et al., where the majority of patients were in the 31-40-year age group.^12,13^

The mean pituitary height in our study group was 4.89 +/- 1.33 mm which is similar to the study by Suzuki et al. but comparatively lower than in the study done by Kumar et al., Maskey et al. and Yadav et al.^5,8,14,15^ However, the mean pituitary height and volume were highest in the 11-20-year age group, aligning with the findings of Shajil et al.^12^ Conversely, Kato et al. reported that pituitary height is a significant parameter, with its peak occurring in the first half of the third decade in women.^16^

The rise in pituitary height during puberty is linked to increased Luteinizing Hormone (LH) production.^5^ The pituitary height showed variations with age correlating with previous studies, the differences between males and females within individual age groups were not statistically significant.^8^ This suggests that while absolute size differences exist, they may not be clinically relevant when assessing individual patients. Kumar et al. reported an increase in pituitary height in females aged 50-59, which was not observed in our study.^5^ This might be due to increase in gonadotropic hormone levels as a result of decreased feedback effect in females in fifth and sixth decades, because of age-related decrease in circulating gonadal steroids hormones.^17^

In our study, females had slightly higher mean pituitary volumes (288.06 +/-111.36 cc) than males. This is consistent with the findings of Singh et al while it contrasts with the findings of Shajil et al.^12,18^ Additionally, the women in the reproductive years have higher mean pituitary height and volume than others.

One of the strengths of this study is its large sample size, encompassing individuals across all age groups, which enhances the generalizability of our findings. Additionally, the use of MRI, the gold standard for pituitary imaging, ensures accuracy in volumetric and dimensional measurements. Moreover, this study provides normative data specific to the South Asian population, filling a critical gap in the literature. However, certain limitations should be considered. The study was conducted at a single tertiary care center, which may not fully represent the general population. Furthermore, hormonal levels were not assessed, which could have provided additional insights into the observed variations.

## CONCLUSIONS

This study establishes normative data for pituitary gland dimensions and volume across different age groups in the general population undergoing MRI. This enables us to discover significant changes in the pituitary gland across a person's lifetime, depending on age and gender. The pituitary height and volume correspond to physiological neuroendocrine variations. This occurs across all ages and genders. Any aberrant variation in the pituitary gland's dimensions and size can help identify any pathology and make an early diagnosis. The findings of this study indicate variations in pituitary height and volume with age, with peak values observed in the second decade of life. These normative values provide a crucial reference for identifying abnormal pituitary morphology in clinical practice. As a result, MRI can be an effective tool for correctly measuring pituitary size, correlating findings with age and gender, and identifying any abnormalities.
